# The Significance of Rhabdomyolysis Secondary to Hypothyroidism

**DOI:** 10.7759/cureus.69715

**Published:** 2024-09-19

**Authors:** Bidisha Baral, Sandesh R Parajuli, Harvey B De Nieva, Laxman Wagle, Hom Nath Pant

**Affiliations:** 1 Internal Medicine, Ascension Saint Agnes Hospital, Baltimore, USA; 2 Internal Medicine, Tower Health Reading Hospital, Reading, USA

**Keywords:** acute kidney injury, drug compliance, elderly female, hypothyroidism, rhabdomyolysis

## Abstract

Rhabdomyolysis is a rare but serious complication of hypothyroidism, typically associated with precipitating factors such as medication interactions, strenuous exercise, and illicit drug use. We present a unique case of rhabdomyolysis in an 89-year-old female due to severe hypothyroidism without identifiable precipitating factors. Laboratory results revealed markedly elevated creatine kinase (CK) levels and acute kidney injury (AKI). The diagnosis was confirmed by critically elevated thyroid-stimulating hormone (TSH) and low free thyroxine (FT4) levels. Prompt initiation of levothyroxine supplementation and fluid resuscitation led to clinical improvement and downward trend in creatinine and CK levels. This case highlights the importance of considering hypothyroidism in the differential diagnosis of rhabdomyolysis and the need for timely T4 supplementation and supportive care to prevent severe complications.

## Introduction

Thyroid function abnormalities are common endocrine disorders [1]. The clinical manifestations, particularly in hypothyroidism, are often non-specific. Muscle symptoms may sometimes be the primary or only clinical manifestation of hypothyroidism, complicating differential diagnosis with other myopathies [2]. Rare muscular manifestations linked to hypothyroidism include rhabdomyolysis, myoedema, acute compartment syndrome, Hoffman's syndrome, and Kocher-Debré-Sémélaigne syndrome [2]. Skeletal muscle necrosis leading to rhabdomyolysis in hypothyroidism is extremely rare and usually associated with precipitating factors such as statins, severe exercise, or illicit drugs [3]. We report a unique case of rhabdomyolysis in an elderly female due to severe hypothyroidism following medication non-adherence and without inciting factors including medications, exercise, and illicit drug use.

## Case presentation

An 89-year-old female with a medical history of hypothyroidism, chronic kidney disease (CKD) stage III, and hypertension presented with a one-week history of generalized weakness that progressed to the extent of requiring assistance for ambulation. Accompanying symptoms included urinary incontinence, decreased appetite, and loose, watery bowel movements without melena or hematochezia. Notably, she denied experiencing pain, focal weakness, asymmetrical limb swelling headache, hematuria, oliguria, and anuria. She also denied any recent fall or trauma. No chest pain, shortness of breath, cough, fever, or chills were mentioned. Recent weight changes, change in voice or neck swelling were not noted. Despite being prescribed levothyroxine and hydrochlorothiazide, she was not compliant with her medications. No new medications were prescribed. She lived by herself and, as per her daughter, was independent with activities of daily living until the recent event. 

Diagnostic assessment

On admission, her vitals were as follows: afebrile with temperature of 36.3°C, heart rate of 60 beats per minute, respiratory rate of 18 breaths per minute, and a blood pressure of 89/53 mm Hg. Physical examination revealed dry mucous membranes with no other remarkable physical findings. The pertinent positive laboratory values on the day of admission are illustrated in Table 1 below. 

**Table 1:** Laboratory results BUN: Blood urea nitrogen; AST: Aspartate aminotransferase; CK: Creatine kinase

Test	Result	Normal Range
BUN	78 mg/dL (27.9 mmol/L)	10-20 mg/dL (3.6-7.1 mmol/L)
Creatinine	8.7 mg/dL (769 µmol/L)	0.6-1.1 mg/dL (53-97 µmol/L)
AST	116 U/L	5-34 U/L
CK	43,712 U/L	29-168 U/L

Urinalysis suggestive of myoglobinuria can be derived from Table 2 below.

**Table 2:** Urinalysis results

Test	Result
Occult Blood	3+
Red Blood Cells	None Detected

On review of records, baseline creatinine was found to be in the range of 1.1-1.3 mg/dL. Retroperitoneal ultrasound of kidney and bladder did not reveal any obstructive pathology. 

Treatment

Initial management involved administering two liters of Lactated Ringer's as bolus. Further evaluation revealed a critically elevated thyroid-stimulating hormone (TSH) level of 99.266 µIU/mL (normal range: 0.5-5 µIU/mL) alongside a markedly low free thyroxine (FT4) level of less than 0.42 ng/dL (<5.4 pmol/L) (normal range: 0.7-1.5 ng/dL; 9-19 pmol/L). With the initiation of thyroid hormone supplementation (levothyroxine 150 mcg) on day one and continuous fluid resuscitation, both creatinine and creatine kinase (CK) levels showed a downward trend. Home health services were arranged, and the patient's daughter confirmed that she will assume responsibility for overseeing medication administration.

On review of records from her post-hospitalization primary care visit, her creatinine at one week was 1.46 mg/dL, which was close to her baseline. Her TSH level at 8 weeks was 6.4 µIU/mL.

**Figure 1:**
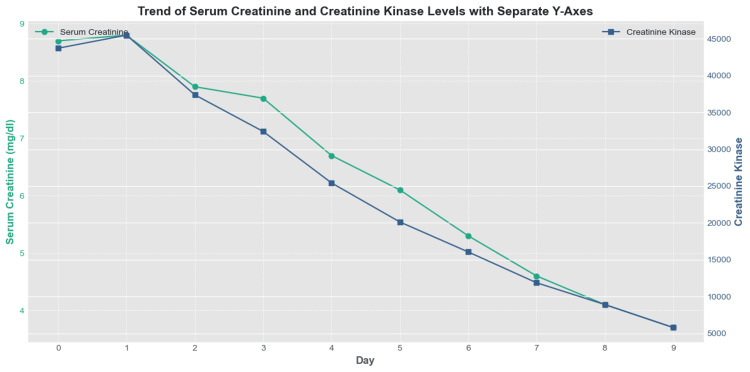
The graph illustrates the trend of creatinine in relation to CK levels over the course of treatment. CK: Creatine kinase

The diagrammatic representation of the response to therapeutic intervention during hospital is below (Figure 1). 

## Discussion

Rhabdomyolysis is an uncommon occurrence in patients with poorly managed hypothyroidism, often triggered by events like exercise, illness, or medication interactions [4,5]. Non-adherence to medication is a known risk factor for rhabdomyolysis in hypothyroid patients, with non-adherence rates reaching 25-50% [6,7]. Ghayur et al. documented a case where non-adherence to T4 treatment in a hypothyroid patient led to acute kidney injury (AKI), requiring hemodialysis for four weeks [8]. Typically, rhabdomyolysis occurs in patients with a history of hypothyroidism; however, in rare instances, it may be the initial presenting symptom, increasing the likelihood of misdiagnosis [9]. Up to 50% of rhabdomyolysis patients might not exhibit clear symptoms [9]. These patients have elevated serum CK and myoglobin levels, though no direct correlation has been established between the severity of hypothyroidism and CK levels [9]. Janjua et al. proposed including hypothyroidism in the differential diagnosis for rhabdomyolysis [5]. The American Thyroid Association recommends screening for hypothyroidism in patients with elevated CK and/or lactate dehydrogenase within two weeks [10]. The most accepted hypothesis suggests that hypothyroidism leads to decreased production of triiodothyronine (T3), impaired glycogen utilization, reduced adenosine triphosphate (ATP) hydrolysis, and diminished mitochondrial activity, resulting in metabolic dysfunction [11]. Thyroid hormone deficiency alters glycogenolysis, mitochondrial oxidative metabolism, and triglyceride turnover, transforming fast-twitch (type II) muscle fibers into slow-twitch (type I), reducing actin-myosin contractility, myosin ATPase activity, and ATP conversion rates, leading to insufficient muscle perfusion, hypoxia, and reduced energy storage [12].

Other rare manifestations of untreated hypothyroidism include Hoffman syndrome, characterized by muscle weakness and calf pseudohypertrophy in adults, and Kocher-Debré-Sémélaigne syndrome, observed in children presenting with growth delay and muscle pseudohypertrophy [13]. Instances of rhabdomyolysis have also been reported in this condition [14].

In patients presenting with AKI secondary to rhabdomyolysis, fluid resuscitation is critical. An initial infusion rate of 400 ml/hour is recommended, aiming to maintain urine output at 1-3 ml/kg/hour [15]. Consider renal-replacement therapy for persistent hyperkalemia above 6.5 mmol/L, symptomatic rapid rises in serum potassium, oliguria, anuria, volume overload, or resistant metabolic acidosis (pH <7.1) [16]. After initiating levothyroxine treatment, normalization of free T3 (FT3), FT4, and TSH levels is typically seen, alongside a decrease in CK levels. TSH and FT4 should be monitored every four to six weeks to assess treatment efficacy. If TSH levels remain elevated, the levothyroxine dosage may be increased by 25-50 µg/day until the treatment target is achieved [17]. As the primary disease improves following fluid infusion and symptomatic treatment, CK levels should normalize, emphasizing the importance of timely T4 supplementation and supportive care [17]. Delayed diagnosis can lead to severe complications requiring renal replacement therapy and higher-level care, including mechanical ventilation [17].

## Conclusions

This case report emphasizes the critical importance of therapeutic adherence in the management of hypothyroidism and illustrates the potential severity of muscle complications in this patient population. Untreated hypothyroidism can lead to potential life-threatening complications, including rhabdomyolysis and AKI. Timely diagnosis is crucial, as delayed diagnosis can result in serious outcomes, necessitating advanced interventions such as renal replacement therapy and mechanical ventilation. Initiation of appropriate treatment, including levothyroxine supplementation and fluid resuscitation, is crucial in preventing further complications and promoting clinical recovery. Furthermore, this case emphasizes the importance of regular monitoring of thyroid function and medication adherence, with a specific focus on elderly patients with hypothyroidism, to prevent such rare but serious complications.
